# Wounds of the Knee-Joint

**Published:** 1918-10

**Authors:** 


**Gunshot Wounds of the Knee-Joint.** Major RICHARD CHARLES, R.A.M.C. *British Medical Journal,* June 29, 1918.

The experience of the author proves conclusively the correctness of the advice given in 1915 by Colonel Gray that conservatism in gunshot wounds of the knee should be the rule. All such injuries should be operated upon within the first 24 hours after a preliminary X-ray which determines the position of a foreign body as well as the extent of bone injury. The operation should be done in a bloodless field (Esmarch bandage). After sterilizing the skin (shaved, and washed with spirit) with 5 o/o picric acid, the wounds are packed with gauze to prevent leakage of infected fluid unto the skin.

The entire tract should be removed intact down to the joint cavity without permitting instruments to touch this infected area. After making an elliptical skin incision about the wound, the skin is undermined and its edges protected by oiled silk. Then an incision is carried on one side down through all layers into the joint cavity to permit a view of the deep end of the tract. With these landmarks established, the tissues enclosing the whole tract are excised in one piece, and, as both ends of the tract are in view, a minimal loss of fibrous tissue takes place.

When the joint is investigated, instruments and gloves are changed. The original opening is enlarged to allow thorough examination. At times it may be necessary to cut across the patella ligament to get adequate exposure. If the missile is lodged in the joint cavity, it is removed, the joint irrigated and closed in layers, with the object of getting first intention.

Experience has shown that the protective mechanism of the knee joint versus infection is much better than was thought; in fact it resists infection better than the soft parts, and, therefore, to avoid infection from without should be regularly shut off from these by suturing the joint. If the missile is impacted in bone, its site should be isolated by saline gauze and it should be chiselled out with adjacent bone; a clean-cut incision being made with osteotome and hammer. The joint is then irrigated and closed. Grooved bone injuries, small comminuted fractures, chipping of the bone, are carefully removed or chiselled away.

In mid line of joint these principles are difficult to apply. Severe lesions in this region often penetrate further into the bone than examination reveals, or the surgeon can excise. In these one may be compelled to resect. However, by reflecting patella and employing Carrel Dakin from the start, one can often save the joint. When the bone lesion is small and the conditions otherwise favorable, the infected area may be curetted away and the joint closed after thorough irrigation.

Another type of wound necessitating departure from the principle of en bloc excision is that with chipping or comminution of the tibial head. Here excision must be practiced in stages. It is necessary to excise first skin wounds and tracts in soft tissues, then reflect patella and excise infected ligamentous structures—usually also the meniscus, and finally, by a vertical incision over the lateral aspect of the head of the tibia, fully expose and chisel away the damaged bone.

In favorable cases the wound is completely closed; otherwise it is dressed with Bipp and packed with gauze, the end of the gauze being brought out through the partly closed wound. When the patella is fractured, the comminution is liable to be so great as to call for complete removal together with neighboring tissues. Precaution must be taken against cutting away any serious membranes or ligaments beyond the margin of necessity. In bringing the parts together it will often be necessary to lengthen the quadriceps tendon. Less serious patella injuries can be excised with metacarpal saw.

Gaps in the synovial membrane may be repaired by bringing down the membrane from the suprapatella bursa and suturing it in place. In 50 consecutive cases there were three deaths (sepsis). In 20 cases reported in detail in this paper there was, as far as the author knows, but one secondary amputation. The article does not speak of functional results.

